# Supplementary figures and images for: Accounting for sensitivity of latent learning to behavioral statistics with successor representations
Source: PLoS Comput Biol. 2026 Mar 24;22(3):e1014131. doi: 10.1371/journal.pcbi.1014131 (PMC13043058; doi:10.1371/journal.pcbi.1014131)

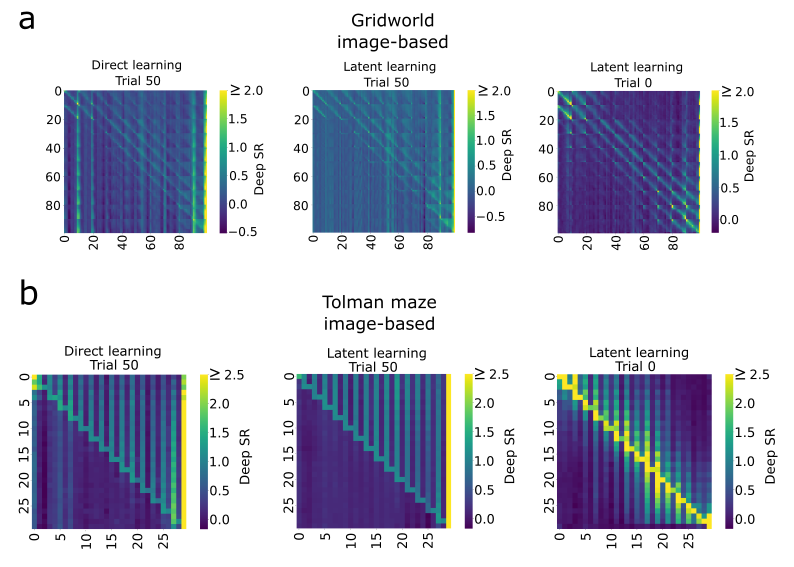

Supplement: S1 Fig — SF for all states are shown for the A) gridworld for the right action and B) Tolman maze for the up action, averaged over 30 simulations. Each row of panels corresponds to the successor transitions of a particular state to every state in the environment. We computed the contribution of each state’s feature to the spatial transition using Eq 11. Trials 0 and 50 correspond to before and after the learning phase, respectively. The SF shows that in both mazes the latent learning agent has learned the local connectivity of the maze during the pre-exposure phase before the learning phase commences. At the end of the learning phase, both agents exhibit similar transition patterns that reflect a movement from the start to the goal location in both mazes. (TIFF) [file pcbi.1014131.s001.tiff]

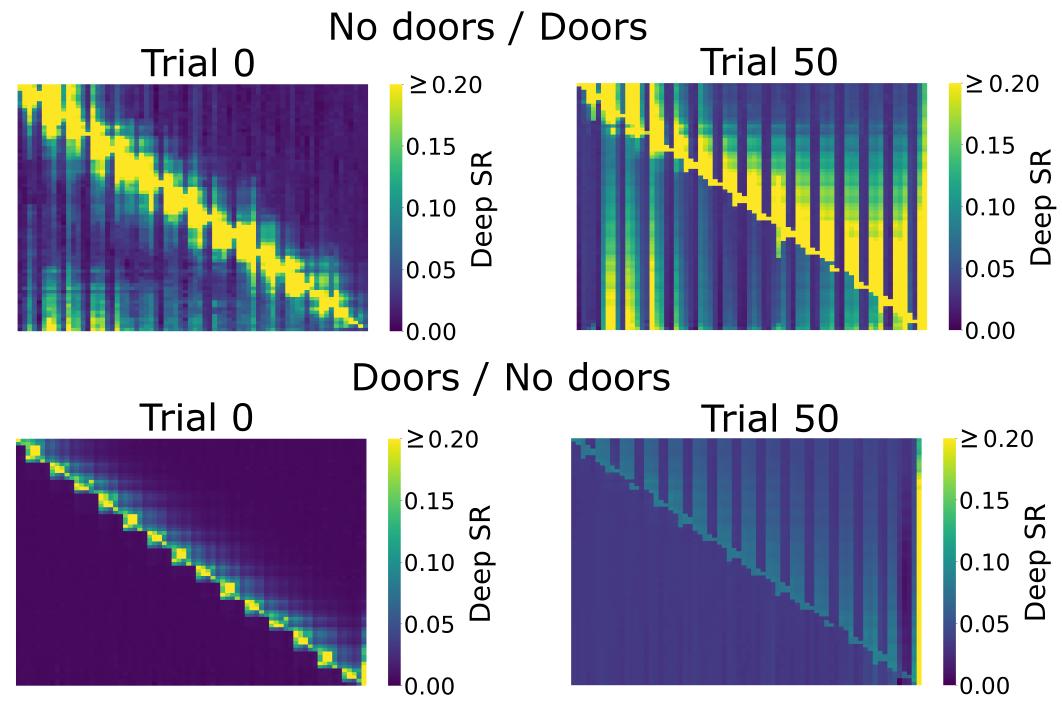

Supplement: S2 Fig — Top: Deep SR of agents under No doors/Door exploration strategy. Bottom: Deep SR of agents under Doors/No doors exploration strategy. Trials 0 shows the path transition differences before the learning phase. The strategy with no doors during the learning phase shows a more concise path transition compared with the one that adopts doors. (TIFF) [file pcbi.1014131.s002.tiff]

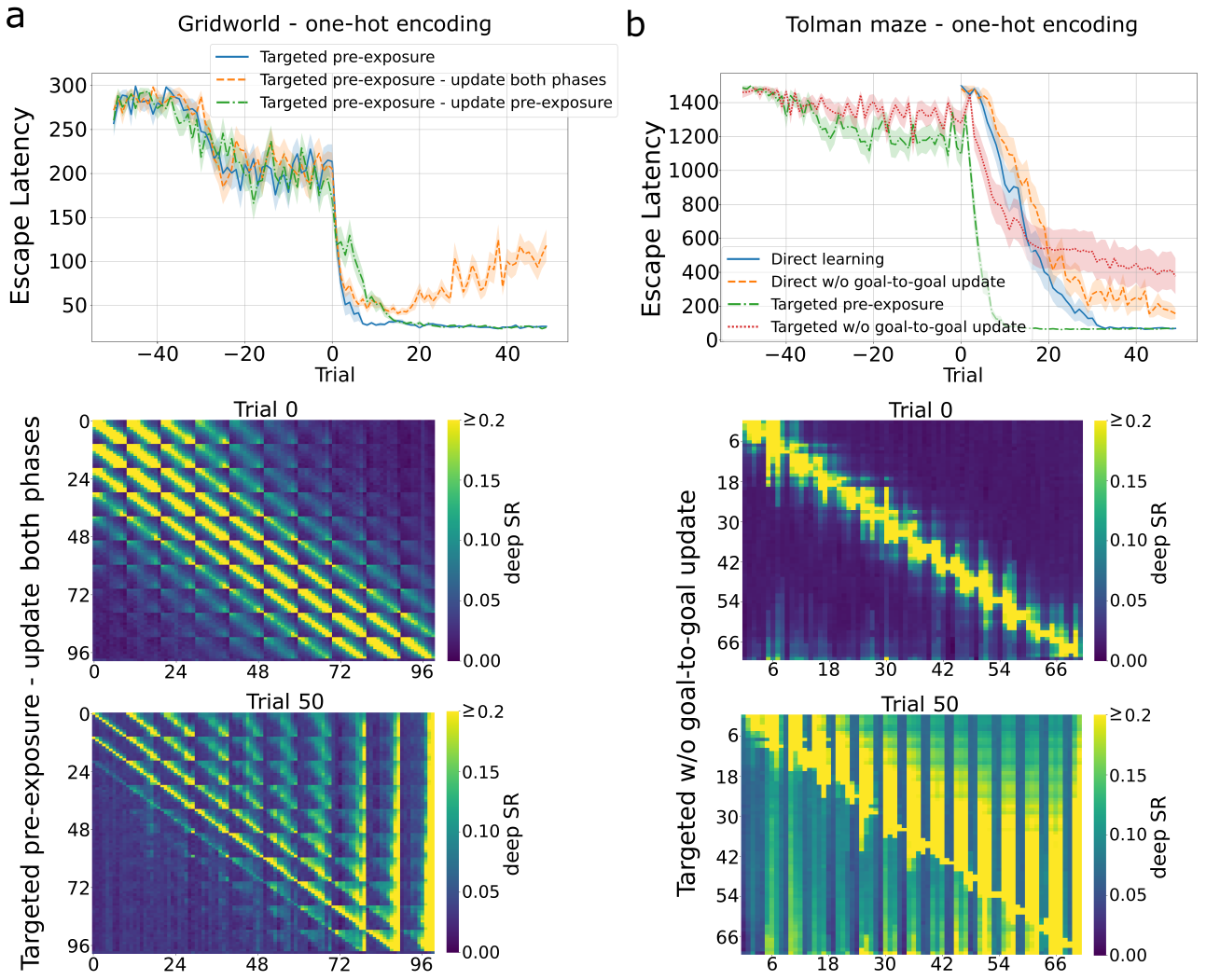

Supplement: S3 Fig — A) The goal state’s SF is trained to match a zero vector of equal dimensionality in gridworld. Top: escape latency indicates that training the goal state with zero vectors during both phases leads to a clear performance drop (orange line, average of 30 simulations, error bars are S.E.M.), whereas applying zero-vector updates only during pre-exposure results in slower learning (green line) compared to the condition without zero-vector updates (blue line). Bottom: Deep SR of the agent when the goal state is updated with zero vectors after pre-exposure (trial 0) and after the learning phase (trial 50). B) Goal-to-goal update effect is switched off in Tolman maze. Top: escape latency shows that direct and latent learning without goal-to-goal updates (orange and red lines, respectively, average of 30 simulations, error bars are S.E.M) is slower and less stably than their counterparts (blue and green lines, respectively). Despite that, latent learning without the goal-to-goal update still emerges earlier than direct learning (trial 10). Bottom: deep SR of the latent learning agents without goal-to-goal updates after pre-exposure (trial 0) and the learning phase (trial 50). (TIFF) [file pcbi.1014131.s003.tiff]
